# Green, Sustainable, and Multifunctional Biobased Hybrid Nanocomposites: Semiconducting Materials with Tunable Molecular Interfaces for Photocatalysis

**DOI:** 10.3390/ijms27073236

**Published:** 2026-04-02

**Authors:** Lalita Chopra, Muskan Thakur, Domenico Pirozzi, Filomena Sannino

**Affiliations:** 1 Department of Chemistry, UIS, Chandigarh University, Gharuan 140413, Punjab, India; lalita.e2341@cumail.in (L.C.); thakurmusu253@gmail.com (M.T.); 2 Department of Chemical Engineering, Materials and Industrial Production (DICMaPI), Laboratory of Biochemical Engineering, University of Naples “Federico II”, Piazzale Tecchio 80, 80125 Naples, Italy; dpirozzi@unina.it; 3 Department of Agricultural Sciences, University of Naples “Federico II”, Piazza Carlo di Borbone I, 80055 Portici, Italy

**Keywords:** biobased semiconductors, heterojunction photocatalysts, multifunctionality, band engineering

## Abstract

Biobased hybrid semiconducting composites are attracting significant attention as sustainable alternatives to traditional inorganic photocatalysts for environmental remediation and energy-related applications. Recent research progress in biobased hybrid photocatalytic systems is critically reviewed to outline their design strategies, photocatalytic mechanisms, and environmental applications. These composites integrate bioderived polymers with metal oxide semiconductors, forming hybrid architectures that improve interfacial contact at the molecular level, enhance charge transfer efficiency, and impart higher structural flexibility. The polymer matrix not only provides mechanical adaptability and functional surface groups, but also serves as an environmentally friendly support that can modulate surface electronic states and influence the photoinduced electron–hole dynamics in the inorganic phase. By controlling the molecular interactions between the polymer chains and metal oxide surfaces, these hybrids can mitigate key limitations of conventional metal oxides, such as rapid electron–hole recombination and restricted visible-light absorption. This review first summarizes the fundamental electronic and structural properties of widely employed metal oxide semiconductors and highlights their intrinsic limitations in photocatalytic processes. It then examines the role of biopolymers from the perspective of molecular structure, charge transport pathways, and interfacial interaction mechanisms with the inorganic component. Various synthesis strategies—including sol–gel, hydrothermal, in situ nanoparticle generation, green synthesis, and surface functionalization—are discussed, with emphasis on their ability to tune the nanoscale morphology and interfacial chemistry of the hybrids. Applications of these biohybrid systems in dye degradation, pharmaceutical pollutant removal, heavy metal reduction, and antimicrobial photocatalysis are analyzed alongside mechanistic insights into charge separation efficiency and band alignment at the molecular interface. Furthermore, challenges related to long-term stability, reproducibility, scalability, and performance in real wastewater matrices are also addressed. Overall, this review provides a thorough discussion on the design principles, photocatalytic mechanism, and environmental applications of biobased hybrid semiconductors, while emphasizing future opportunities for the development of efficient and sustainable photocatalytic systems.

## 1. Introduction

A semiconductor is a material with electrical conductivity between that of a conductor and an insulator. This nature allows it to regulate electrical charge when the right conditions are met. Providing enough energy, electrons in a semiconductor can go up from valence to conduction band, creating electron–hole pairs that are involved in electrical or catalytic activities [1]. Electronic and optoelectronic devices, catalysis, and sensing applications are the main sectors for using these materials [2]. Despite the technological progress achieved with conventional semiconductors, issues related to material sustainability, fabrication cost, and environmental persistence have encouraged the exploration of alternative design strategies. The identification of the properties of a semiconductor as the result of decades of research has led to the development of the design of modern photocatalytic materials [3]. These discoveries form the foundation for developing eco-friendly biohybrid semiconductors that combine the functionality of inorganic semiconductors with the natural beauty of biopolymers. Semiconductors are of two main types: elemental semiconductors and compound semiconductors. Elemental semiconductors, like pure silicon, gallium, or germanium, are commonly used in electrical and electronic devices. Compound semiconductors, such as metal oxides and metal sulfides, are often used in photocatalytic processes [4]. When a semiconductor is in its pure form, without any added substances, it is called an intrinsic semiconductor [5]. However, when small amounts of other elements are added to change its electrical behavior, it becomes an extrinsic semiconductor. Doping in semiconductors can be of two types, p-type and n-type, depending on whether the added impurity is an acceptor or a donor [1]. In compound semiconductors, a slight imbalance in the ideal chemical ratio, known as a stoichiometric defect, can lead to the formation of donor or acceptor sites [6]. Certain d-block metal oxides, such as ZnO, Fe_2_O_3_, and TiO_2_, typically behave as n-type semiconductors due to a minor deficiency in oxygen atoms in their crystal lattice. On the other hand, compounds like FeS and Cu_2_O tend to act as p-type semiconductors because of the presence of metal vacancies within their lattice structures [7]. Based on bandgap theory, semiconductor materials with a bandgap in the range of 1 eV to 4 eV are ideal for photocatalytic applications, as this range allows them to absorb visible or near-UV light efficiently. In addition to the bandgap, the mobility of charge carriers and the positions of the band edges also play a critical role in determining the photocatalytic performance of a semiconductor material [6].

Due to the remarkable properties of semiconductors, like band structure, tunable physical and chemical properties, they emerged as promising materials in photocatalysis [8]. Photocatalysis in the presence of semiconductors is achieved by the excitation of electrons from the valence to the conduction band when subjected to light radiation [9]. Upon light irradiation with energy equal to or greater than the bandgap, electrons are excited from the valence band to the conduction band, generating electron–hole pairs [10], and further, these charge carriers take part in multiple redox reactions at the surface of semiconductors to degrade pollutants, splitting water and distinct organic reactions. The development of a photocatalytic process is required due to two crucial issues: the increase in environmental pollution and the need for sustainable energy. Concern has been raized due to the rapid increase in industrialization and urbanization. Various types of industries are responsible for the discharge of pollutants into the environment. Although numerous traditional methods are reported to treat wastewater, they become inefficient and cannot remove pollutants from the environment [11]. By considering all these, researchers have reported semiconductor photocatalysts that are becoming suitable material for environmental remediation [12]. One of the most explored applications of semiconductors is the degradation of various pollutants such as dyes, drugs, organic compounds, and pesticides. Various semiconductors have been reported by researchers, which have shown remarkable efficiency in the degradation of harmful compounds into simpler molecules. In the photocatalytic degradation process, reactive oxygen species are generated to degrade the pollutants. These species are hydroxyl radicals, superoxide anions, and oxygen radicals, which are responsible for breaking down the moieties into simpler molecules [13]. The concept of photocatalysis was introduced in 1964 by Doerfler and Hauffe [14], who reported photoassisted carbon monoxide (CO) oxidation using a ZnO catalyst. Later, in 1972, Fujishima and Honda [15] made a significant breakthrough by demonstrating the photoelectrolysis of water using a TiO_2_ electrode under light irradiation. These studies attracted the curiosity of the scientific community, becoming a source of energy, catalysis, and environmental remediation through the photocatalytic processes [16]. Among the semiconductor materials of photocatalytic applications, anatase TiO_2_ [17] and wurtzite ZnO [18] have been the most widely investigated due to their appropriate bandgaps and low charge carrier recombination rates. A literature survey revealed that numerous metal oxide semiconductors, such as SrO_2_, ZrO_2_, CeO_2_, CdO_2_, and CuO, exhibit excellent photocatalytic efficacy for the degradation of multiple organic pollutants, including drugs, dyes, and pesticides [19,20]. Each semiconductor material is different in terms of its properties, like bandgap, charge carrier transport, surface area, stability, light response, and recombination rate. For an efficient photocatalyst, one must have an optimized bandgap in visible light from 2 eV to 3 eV [21], a high surface area, and a low recombination rate.

Apart from this, semiconductors are playing their role in other crucial processes like the generation of hydrogen, the reduction of carbon dioxide, and antimicrobial activities, which can be applied in hospitals, food packaging, and textiles [6]. With these innovations, semiconductor photocatalysts have become a pathway towards sustainable energy creation and a cleaner environment. The importance of semiconductor photocatalyst lies in giving environmentally friendly, sustainable, and cost-effective remediation to many global issues [22]. In comparison to other conventional methods, semiconductor photocatalysts can degrade harmful pollutants completely by releasing harmless products. This invention has become an attractive method for wastewater treatment and environmental solutions. Also, they are capable of utilizing solar energy in other processes, like water splitting to produce hydrogen and the reduction of carbon dioxide [23]. Further, antimicrobial properties have shown their capability in healthcare, food packaging, and other materials [24]. Even so, conventionally established inorganic semiconductor photocatalysts have shown significant progress. There are emerging challenges associated with these non-biodegradable materials, like limited visible-light absorption, poor recyclability, environmental issues, etc. Combining semiconductor and biobased polymeric materials has become an emerging solution to address these issues by not only upgrading their properties but also making them more sustainable, efficient, and environmentally friendly. Over recent years, the integration of semiconductors and biobased polymeric materials has increased as a potential approach to overcome these limitations by enhancing their properties. The novelty of this review lies in its thorough discussion of biobased hybrid semiconductor systems at the molecular interface. Most traditional reviews mainly dwell on inorganic semiconductor photocatalysts. However, this work shows how biopolymers play a critical role in controlling interfacial charge transfer, providing structural flexibility, and enhancing the photocatalytic efficiency of hybrid architectures. Besides this, the present review critically discusses the synthesis pathways, the structure, property, and performance relationship, and photocatalytic mechanisms involved in the degradation of pollutants and environmental remediation. Along with this, the review thoroughly examines various issues such as the stability, profitability, and performance capacity of real wastewater systems, and at the same time outlines the development of sustainable photocatalytic materials. This review emphasizes biobased semiconducting hybrids and their photocatalytic applications. It focuses on different classes of biobased semiconductors, photocatalytic mechanisms and their applications in environmental remediation, and the current limitations and future prospects for the applicability of sustainable and efficient photocatalytic systems.

## 2. Limitations of Conventional Semiconductor Photocatalysts

From the already existing research on semiconductor photocatalysts, they have shown remarkable potential in various environmental remediation applications like pollutant degradation, abilities in chemical synthesis, energy production, and many more applications, but still they have shown some restrictions in large-scale applications. A significant weakness is that the light absorption of most photocatalysts is limited, and so can only absorb ultraviolet radiation, which comprises 5% of the solar spectrum, with the rest of the visible and infrared light remaining unexploited [25]. Moreover, the high rate of recombination of electron–hole pairs generated upon UV irradiation is a key factor that leads to low quantum efficiency and also slows the general reaction rate of photocatalysts [26]. It has also been noted that a few semiconductors have become unstable in the presence of harsh conditions. Scalability is also an issue, since it is hard to sustain efficiency when using real wastewater systems because they contain various contaminants. pH can vary and be turbid, thereby restricting the penetration of photons. In addition, recovering and recycling nanoparticles from treated water for reuse is a challenge on both an environmental and a cost level. This limitation directly affects the economic feasibility and environmental sustainability of photocatalytic systems, as inefficient recovery and reuse can lead to an increase in operational costs and secondary pollution [12]. Therefore, developing photocatalysts with high reusability and stability over multiple cycles is essential for practical applications. Also, their marketability is also greatly affected by the high costs of their synthesis, the inability to control their morphology, and the lack of standardized testing protocols [27]. Thus, although semiconductor photocatalysts have great potential in laboratory-scale experiments, they undergo considerable changes, like doping, heterojunction, or surface functionalization, to address these limitations and attain viable and sustainable applications [28]. The rigid and brittle nature of purely inorganic semiconductors limits their usability in flexible, multifunctional, and eco-friendly environment platforms [29]. To address this issue, biopolymers have been developed as promising functional components, providing intrinsic ionic transport pathways, stability, flexibility and eco-friendly nature to semiconductor photocatalysts [30]. This indicates the need for understanding the synergism between biopolymers and semiconductor photocatalysts.

## 3. Biopolymers as Functional Components

**Figure 1:**
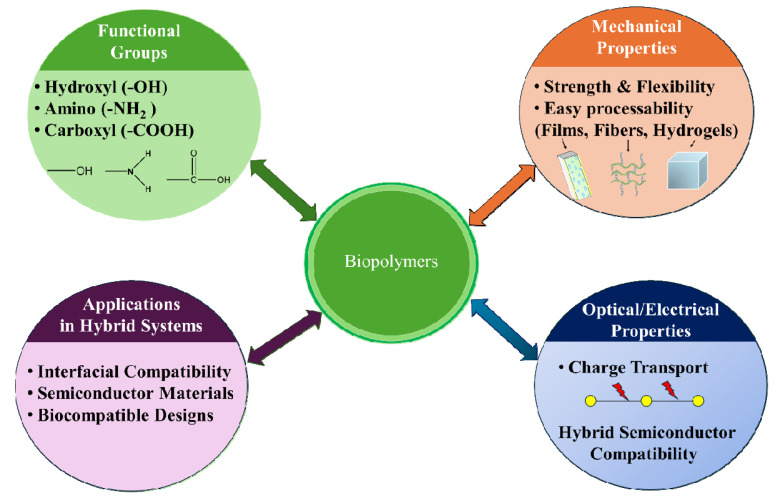
Structural features and functional roles of biopolymers.

Biopolymers are naturally obtained macromolecules with a wide range of functional groups, such as hydroxyl, amino, and carboxyl, enabling chemical modification and strong interactions with inorganic materials [31]. Thus, biopolymers such as cellulose, chitosan, starch, alginate, silk fibroin, polypeptides, etc., have risen as functional materials in semiconductor-based systems due to improved interfacial compatibility within hybrid systems [32]. The structure of biopolymers exhibits a semicrystalline or amorphous nature that influences their mechanical, optical, and electrical properties. Cellulose and chitosan show highly ordered hydrogen-bonded networks, displaying excellent mechanical strength and flexibility. A review by Moon et al. [33] summarized the crystal structure and morphology of cellulose, also confirming that it consists of both crystalline and amorphous regions and highly ordered hydrogen bonds, highlighting its mechanical strength and functional properties. Similarly, Rockwood et al. [34] reported that silk fibroin derived from *Bombyx mori* cocoons demonstrates excellent mechanical properties, biocompatibility, and the ability to easily process into hydrogels, fibers, films, etc., due to its protein structure, making it a potential biopolymer for hybrid material design. Besides structural flexibility, it is the functional groups and molecular arrangement of biopolymers that determine their capability to carry charges, which is crucial for their function in hybrid semiconductor systems. Overall, the structural features and functional roles of biopolymers are illustrated in Figure 1.

### 3.1. Charge Transport Mechanisms

**Figure 2:**
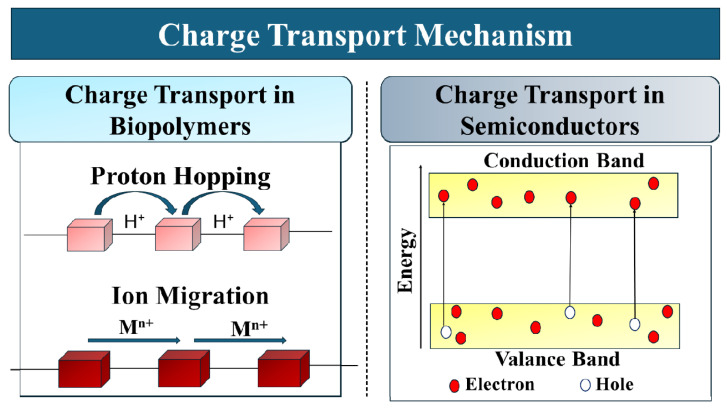
Comparison of charge transport mechanisms in biopolymers and inorganic semiconductors.

Charge transport mechanisms are fundamentally influenced by ionic conduction mechanisms in biopolymers and vary primarily from those in inorganic semiconductors. Proton hopping and ion migration through hydrogen-bonded networks are primarily governed mechanisms in biopolymers [35]. Charge transport behavior is influenced by the structure of biopolymer molecules, their degree of crystallinity, and chemical modification. A rise in crystallinity generally restricts the ionic mobility, and the amorphous nature promotes ion transport through better segmental motion [36]. Inorganic semiconductors show charge transportation mainly through band conduction of electrons and holes [37], whereas biopolymers rely on ionic mobility promoted by their functional groups and hydrogen-bonded networks [38]. In semiconductor–biopolymer hybrid systems, both ionic and transport pathways coexist, facilitating charge transfer [39]. Overall charge transfer efficiency within hybrid systems is improved by chemical doping, blending with conductive fillers, or incorporation of semiconductor nanoparticles, introducing localized electronic pathways. Biopolymers act as charge-mediating matrices in semiconductor–biopolymer composites, suppressing electron–hole recombination by generating interfacial trapping sites and enabling directional charge migration [40]. A comparison of charge transport mechanisms in biopolymers and inorganic semiconductors is shown in Figure 2.

### 3.2. Advantages in Hybrid Systems

Biodegradable and environmentally compatible biopolymers have naturally attracted attention as potential candidates to be used in semiconductor systems. Unlike synthetic polymers and inorganic supports, biopolymers are not only renewable and environmentally friendly, but can also degrade unwanted by-products [41]. In addition to that, biopolymers provide mechanical flexibility and structural stability to the hybrid systems, which can be utilized for flexible devices and multifunctional platforms. The presence of plentiful functional groups in biopolymers promotes the strong chemical bonding and physical adsorption of metal oxide semiconductors [42]. Hence, the resulting dispersion is more uniform, with less nanoparticle accumulation and improved photocatalytic performance [37]. Besides photocatalysis, the biocompatible nature of biopolymer–semiconductor hybrids has led to the development of new strategies in sensors, bioelectronics, and antimicrobial applications [43]. Their ability to operate in aqueous and biologically relevant environments, combined with better charge transport and surface reactivity, makes these hybrid materials promising candidates for next-generation sustainable technologies [44].

## 4. Emergence of Biobased Semiconducting Photocatalysts

In the last few years, the evolution of biobased semiconducting photocatalysts has emerged as the most sustained method to produce metal oxide semiconductors. Although these semiconductors have proved useful in solar energy applications to clean up the environment and convert it into energy, their limitations, which include UV light dependence, photocorrosion, high cost of manufacture, and environmental toxicity, necessitate the exploration of more environmentally friendly alternatives [45]. Biobased semiconductors are extracted from various things like biomass, naturally existing polymers, and some waste products [46]. These extracts act as precursors due to their lower cost, abundant availability, and nontoxic and less hazardous properties. They are well-suited for green chemistry, which makes them the most promising materials for various applications. Biobased semiconductors show distinct physical and chemical properties. Precursors like cellulose, starch, proteins, etc., can synthesize carbon-based semiconductors, whereas plant biomass can produce carbon dots. The environmentally benign and scalable synthesis of biobased photocatalysts is one of its greatest assets. These biobased semiconductors can be produced by various methods, such as pyrolysis [47], sol–gel treatment [48], hydrothermal treatment [49], and others. These methods can also use the precursors extracted from agricultural waste, which tends to be low-cost and contributes to zero-waste technologies [50]. Due to this, they show multiple advantages and have become highly attractive to attain sustainable development goals. The integration of biopolymers and semiconductor photocatalysts has been discussed in many systems, indicating enhanced properties and overall photocatalytic performance. Biopolymers such as chitosan, cellulose, starch, etc., provide better surface interactions with semiconductors and facilitate nanoparticles to disperse uniformly. Recent research demonstrates that integrating biopolymers with semiconductor photocatalysts increases photocatalytic performance significantly due to better absorption properties, increased surface area, and improved interfacial interactions [51]. For example, Chauhan et al. [52] incorporated cellulose and chitosan films with g-C_3_N_4_, and the resulting hybrid films demonstrated around 96–98% efficacy in the removal of dyes and heavy metal ions. Shi et al. [53] used chitosan–cellulose acetate as a biopolymer system with the addition of TiO_2_ as a photocatalyst, and the resulting microspheres supported on cellulose acetate fibers showed better contact between the biopolymer matrix and semiconductor, exhibiting increased adsorption and photocatalytic degradation of methyl orange. Alhassan [40] synthesized MnCo_2_O_4_ nanospinels stabilized with chitosan and cellulose-based biomass, and they demonstrated 96% photocatalytic degradation of methylene blue with enhanced crystallinity and porosity. Collectively, these studies highlight the importance of biopolymers in improving photocatalytic efficiency, pollutant adsorption, and structural stability in hybrid semiconductor systems. Such recent studies outline the benign support of biopolymers and their contribution to synergistic effects in biobased semiconducting photocatalysts.

## 5. Metal Oxide Semiconductors: Key Features

Metal oxide semiconductors are one of the extensively investigated inorganic photocatalytic materials due to their structural stability, tunability, and ability to generate reactive oxygen species (ROS) under light irradiation [19,54]. Metal oxide semiconductors possess inherent optical and electrical properties and wide bandgaps, making them notably applicable for environmental remediation and energy conversion applications [50].

### 5.1. Commonly Used Metal Oxides

Several metal oxides, like ZnO, TiO_2_, SnO_2_, CuO, Fe_2_O_3_, etc. have been widely explored for photocatalytic applications due to strong photocatalytic activity under ultraviolet (UV) light. These metal oxides have gained considerable research interest due to their electronic structure, bandgap energy, and charge carrier dynamics strongly influencing their photocatalytic performance. Özgür et al. [55] reviewed ZnO as an n-type semiconductor, exhibiting a direct bandgap of approximately 3.3 eV and a high exciton binding energy of 60 meV. This contributes to its strong oxidative capability as a UV-responsive photocatalyst. In that review, the authors highlighted the effective photocatalytic degradation by ZnO due to its intrinsic optical and electronic properties. TiO_2_ exhibits three main crystalline forms—anatase, rutile, and brookite—with bandgap energies around 3.0–3.2 eV. The structural stability and non-toxic nature of this metal oxide make it a potential material in photocatalysis [56]. Similarly, Batzil and Diebold [57] reported that SnO_2_ is an n-type semiconductor with excellent chemical stability and electron mobility, making it appropriate for photocatalytic applications, yet the wide bandgap of SnO_2_ is around 3.6 eV, limiting its visible-light absorption, and therefore doping, surface modification, or hybridization techniques are employed to increase the photocatalytic activity. Kay, Cesar, and Grätzel [58] highlighted that hematite, i.e., α-Fe_2_O_3_, shows a bandgap of approximately 2.1 eV and is a visible light-active semiconductor. This makes it a promising applicant for the degradation of photocatalytic pollutants and solar water oxidation [59]. Although the bandgap is suitable for hematite, the authors noted short hole diffusion lengths and rapid charge recombination, which reduce its overall photocatalytic activity. Structural modification and composite formation can be employed to enhance its activity towards photocatalytic degradation. CuO is a p-type semiconductor and more responsive to visible light due to a narrower bandgap than ZnO and TiO_2_—around 1.2–1.7 eV [60]. The charge recombination still exists as a challenge, reducing its photocatalytic activity. There have been various approaches to address this challenge, including doping with metal or non-metal elements, forming heterojunctions with other semiconductors, surface modification, or biobased polymer coupling to enhance charge separation and increase photocatalytic performance [61]. All these metal oxides differ in their bandgaps, charge carrier dynamics, stability, and surface properties, which are the main factors that affect their photocatalytic behavior [8,15]. Therefore, modifying these properties through strategies such as doping, surface modification, and hybridization is often needed to increase their photocatalytic efficiency [45]. Even though these materials have been studied quite intensively, the majority of reports simply mention the degradation efficiency and do not include a full kinetic analysis or give information about the stability of the material over an extended period of time [62]. Thus, it is quite challenging to make a direct comparison of their real-world effectiveness.

As observed in Table 1, commonly used metal oxide semiconductors such as ZnO, TiO_2_, SnO_2_, and α-Fe_2_O_3_ demonstrate favorable properties, including chemical stability and strong oxidative capability. But along with that, many of them suffer from limitations such as wide bandgaps and rapid charge carrier recombination. These limitations outline the need for strategies to modify metal oxide semiconductors with biobased materials to enhance photocatalytic efficiency.

### 5.2. Synthesis Approaches

The ability of metal oxides to perform as catalysts strongly depends on their approach to synthesis. Controlled fabrication of the crystalline nature, particle size, and surface area of the photocatalysts to be prepared can result in features such as increased light absorption, more efficient separation of charges, and hence higher photocatalytic activity. Various synthetic methods have been used to modify these characteristics for their targeted application [63].

#### 5.2.1. Sol–Gel Method

**Figure 3:**
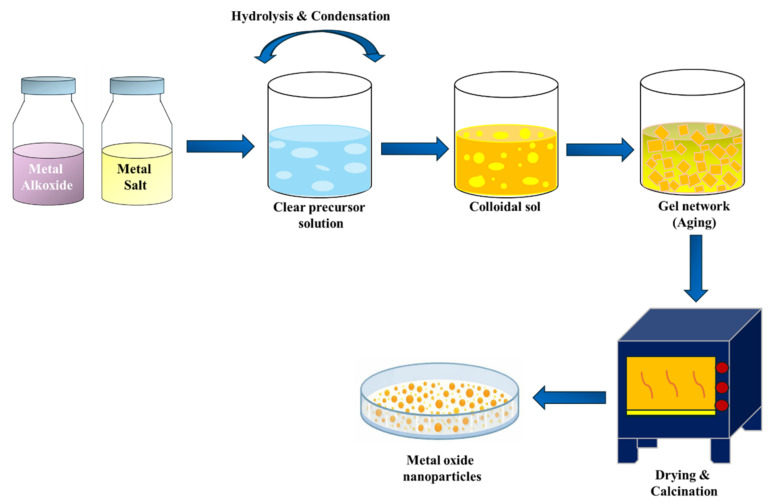
Sol–gel route for the synthesis of metal oxide nanoparticles.

The sol–gel method is considered among the most popular procedures for the preparation of metal oxide photocatalysts due to its simplicity, easy processing, and capability of producing homogeneous nanoparticles with controlled formulation [64]. In this process, metal alkoxide or metal salt precursors undergo hydrolysis and polycondensation reactions and then form a colloidal solution, which later gets modified into a gel and is converted to oxide via controlled drying and calcination. The route for synthesizing metal oxide nanoparticles by sol-gel method is shown in Figure 3. The sol–gel method facilitates accurate particle size, crystallinity, and porosity, which are important for increasing photocatalytic efficiency [65]. Bui et al. [66] synthesized a MgAC–Fe_3_O_4_ metal oxide composite by the sol–gel method for advanced wastewater treatment, demonstrating that the sol–gel technique is flexible in synthesizing hybrid photocatalyst systems with increased activity.

#### 5.2.2. Hydrothermal Method

**Figure 4:**
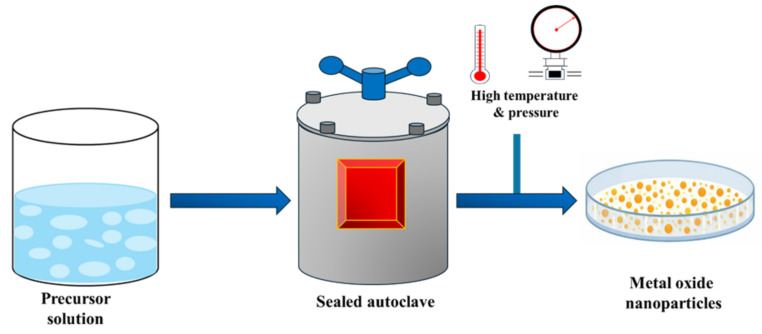
Hydrothermal method for the synthesis of metal oxide nanoparticles.

The hydrothermal technique includes the reaction of precursors at increased temperatures and pressures in a sealed autoclave. The precursor solution used in this method typically consists of appropriate metal precursor dissolved in a suitable solvent, with pH-controlling or structure-directing additives. This method facilitates the production of well-crystallized metal oxide nanostructures without any calcination after synthesis as illustrated in Figure 4. This method leads to the development of a variety of morphologies, like nanorods, nanospheres, hollow microspheres, etc., by modifying reaction conditions such as temperature, duration, and solvent used [67]. The hydrothermal method results in higher crystallinity and larger specific areas than the sol–gel method. Yu et al. [68] prepared hollow Fe_2_O_3_ spheres using controlled hydrothermal reaction, illustrating that the diverse morphologies and properties of the crystal can be achieved using the hydrothermal technique.

#### 5.2.3. Chemical Vapor Deposition (CVD)

**Figure 5:**
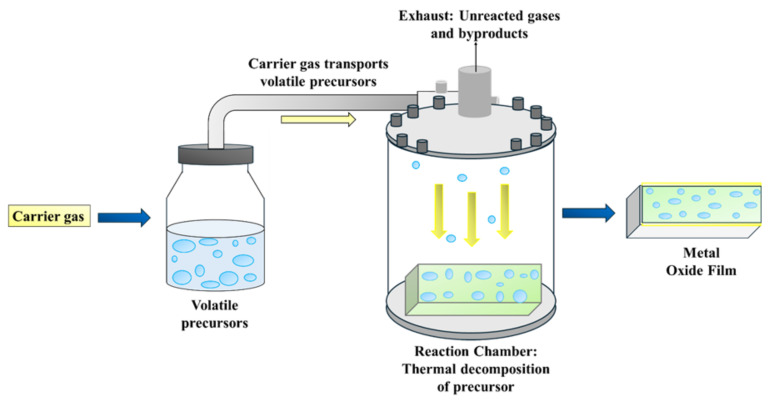
Chemical vapor deposition (CVD) technique for the synthesis of metal oxide films.

Chemical vapor deposition is a vapor-phase technique in which volatile precursors degrade on a heated substrate to synthesize metal oxide films [69]. In this method, precursors are typically volatile metal-containing compounds that are transported in the vapor phase to the reaction chamber using a carrier gas. The chemical vapor deposition technique is shown in Figure 5. This method is less commonly used for preparation in bulk, but it produces uniform thin films and coatings advantageous for photoelectrodes and photocatalysts. This method provides control over film thickness, phase composition, and surface morphology of the synthesized material, making it valuable in designing photocatalytic devices [70]. Barreca et al. [71] synthesized ZnO–TiO_2_ nanocomposites by the chemical vapor deposition method, in which controlled deposition resulted in well-structured nanostructures and improved photocatalytic properties.

#### 5.2.4. Green Synthesis

Green synthesis employs biological agents, such as plant extracts, microorganisms, and biopolymers, as reducing and stabilizing agents to synthesize metal oxide nanoparticles under mild and environmentally friendly conditions. This method reduces the use of hazardous chemicals and lowers energy consumption [72]. Swain et al. [73] reviewed the use of plant extract-mediated methods to produce ZnO nanoparticles with controlled size and morphology, achieving significant photocatalytic degradation of organic pollutants in wastewater. In another review by Mallah et al. [74], green synthesis of metal oxide nanoparticles was highlighted, along with their application in removing toxic dyes from industrial water.

#### 5.2.5. Morphology Control

The photocatalytic behavior of metal oxides is strongly influenced by their morphology by affecting the surface area, active site exposure, and light absorption. Researchers alter the synthesis parameters, including precursor concentration, pH, temperature, and reaction time, to produce a wide range of nanostructures, such as nanowires, nanorods, hollow spheres, nanosheets, and nanoparticles [75]. Larger surface areas increase the number of active sites for redox reactions, offering distinct advantages for photocatalysis [76]. Effective control of morphology combined with synthesis strategies leads to better charge separation and transport, which are necessary for efficient photocatalytic degradation of pollutants and other reactions [77]. Ahmed et al. [75] evaluated the influence of morphological evolution of ZnO nanostructures and synthesis conditions, like precursor ratios and reaction time, to enhance photocatalytic degradation, due to an increase in surface area and improved charge separation.

### 5.3. Limitations of Standalone Metal Oxides

Standalone metal oxide photocatalysts, regardless of their promising intrinsic properties, exhibit several intrinsic limitations that obstruct their practical use in environmental and energy systems. Limited utilization of the solar spectrum is one of their most significant limitations, arising due to wide bandgaps [62]. Also, these materials suffer from rapid recombination of photogenerated electron–hole pairs, which disperses the charge carriers before participating in surface redox reactions and lowers their photocatalytic performance [78]. Additionally, metal oxide nanoparticles show mechanical drawbacks like brittleness and poor flexibility, complicating their integration into flexible substrates and continuous flow systems [79]. Even with high chemical stability, surface modification is frequently needed to tailor band structures, improve light harvesting, and suppress recombination, which adds a layer of synthesis complexity and cost. Liang et al. [80] outlined that the pristine TiO_2_ requires heterojunction formation, doping, or draft engineering to extend its photoresponse into the visible region, highlighting the need for structural modifications to overcome its intrinsic limitations. In a paper by Zaki et al. [81], the limitations, such as surface agglomeration and low specific surface area, exhibited by bare metal oxides were outlined, leading to a reduction in the number of accessible reaction sites and lower adsorption of reactants at active interfaces. Collectively, these limitations emphasize the need for composite formation, surface engineering, and incorporation of other functional materials to achieve better absorption of visible light, efficient charge separation, mechanical flexibility, and enhanced long-term cycling stability for real-world applications.

### 5.4. Factors Affecting Photocatalytic Activity

The use of semiconductor materials in photocatalysis is governed by a range of physicochemical factors. One of the crucial factors is the bandgap energy, which determines the wavelength of light that can be absorbed to generate electron–hole pairs. Semiconductors with appropriate bandgaps are capable of making the most of solar energy for photocatalysis [27]. These physicochemical traits are fundamentally controlled by structural features of materials that include surface functional groups, porosity, and defect states [79,81]. These factors influence the electronic properties, such as bandgap energy and charge carrier dynamics, which in turn determine the photocatalytic properties [78]. Surface features, including surface area and shape, also contribute to catalysis efficiency. Nanomaterials with larger surface areas can support more adsorption and redox reaction sites [82]. On the other hand, the electron–hole recombination rate is a parameter that greatly affects the photocatalytic effectiveness, because faster recombination means fewer charges are left for the oxidation and reduction processes [56]. The catalyst amount, acidity or alkalinity of the solution, initial concentration of pollutants, and luminance are some other parameters that can affect the extent of pollutant decomposition. Hence, tuning these parameters is very important for semiconductor-based hybrid systems to deliver high photocatalytic performance in environmental remediation.

## 6. Hybrid Biopolymer–Metal Oxide Systems

The synergism of biopolymers with metal oxide semiconductors has emerged as a powerful approach to overcome the limitations associated with standalone inorganic photocatalysts and achieve multifunctional performance, integrating environmental compatibility with enhanced photocatalytic efficiency. To design such hybrid systems, careful consideration of synthesis strategies, interfacial engineering, and structural tuning is required to introduce effective charge transfer and mechanical integrity.

### 6.1. Design Strategies

Hybrid biopolymer–metal oxides are constructed primarily by two design frameworks, i.e., physical blending and in situ synthesis. Physical blending is a process of merely mixing a biopolymer matrix with preformed metal oxide nanoparticles. It is very easy to fabricate and is mechanically stable, but lacks the bonding at the interface to a great extent. On the other hand, synthesizing metal oxides in situ within a biopolymer network leads to stronger interfacial bonding, as well as better dispersion and uniformity [83]. This in turn facilitates enhanced charge transfer across the hybrid interface [84]. Hamden et al. [85] prepared TiO_2_ nanoparticles in situ within a chitosan matrix using the sol–gel method. The study revealed that the improved dispersion of nanoparticles and the photocatalytic activity were due to chitosan, which also acts as a template for TiO_2_ crystal growth control. Besides that, surface modification of polymers by methods such as carboxylation, amination, or the addition of conductive species has resulted in better polymer and metal oxide surface compatibility and strengthened interfacial charge transfer, thus reducing charge recombination rates [86]. Layer-by-layer (LbL) deposition and successive nanocomposite formation are examples of innovative assembly methods that are increasingly being recognized. They make it possible to tailor the stacking of metal oxide phases and polymeric layers for directional charge migration and an enlarged active surface area [87].

### 6.2. Structure–Property Relationships

The structure–property relationship in hybrid biopolymer–metal oxide systems plays an important role in understanding and optimizing performance. The biopolymer matrix not only improves mechanical flexibility and eco-compatibility but also plays a primary role in influencing the morphology and charge dynamics of inorganic nanostructures. Zhang et al. [88] demonstrated that nanocrystalline cellulose (NCC) templates regulate the growth and dispersion of ZnO nanoparticles on a carbonized biochar matrix. This leads to increased surface area in composites, improved light absorption, and better separation of photogenerated electrons and holes compared with pristine ZnO, which collectively facilitated synergistic adsorption-photocatalytic degradation of methylene blue. A similar study on biochar–ZnO nanocomposites by Yu et al. [89] revealed that increased porosity and extended surface area facilitate more effective contact between pollutants and active sites on metal oxide surfaces, while the carbonaceous matrix can also support carrier transport and lower recombination rates. ZnO–biochar derived from biomass has demonstrated that a porous carbon framework provides numerous adsorption sites and pathways for charge separation, thereby increasing photocatalytic degradation under visible-light irradiation [90]. Overall, these findings highlight the importance of interfacial architecture between biopolymer and metal oxide phases in determining the effectiveness of hybrid photocatalysts. However, the majority of the reported studies only emphasize improved degradation efficiency and neglect charge transfer kinetics or long-term working stability systematically [91]. Therefore, they hardly promote a profound comprehension of the structure–performance correlations. In conclusion, the photocatalytic performance of these hybrid systems depends on the inherent connection between the material structure, their electronic properties, and their catalytic performance [92]. For example, surface functional groups and porosity help in pollutant adsorption and provide more active sites, whereas heterojunction architecture and interfacial design affect band structure and help in charge separation. In this manner, material structure immediately controls electronic behavior, which determines the photocatalytic performance [61]. New developments in hybrid material design, including dynamic covalent networks, hierarchically porous architectures, and multifunctional nanocomposites, clearly illustrate that exact molecular and structural level control can lead to a significant increase in material functionality. These methods strengthen materials, provide them with more exposed surface area, and also enable the creation of more effective charge transfer pathways [93]. Adopting these design methods in biobased photocatalytic systems would not only enhance their performance but also extend their use in environmental remediation.

### 6.3. Charge Transport and Band Engineering

**Figure 6:**
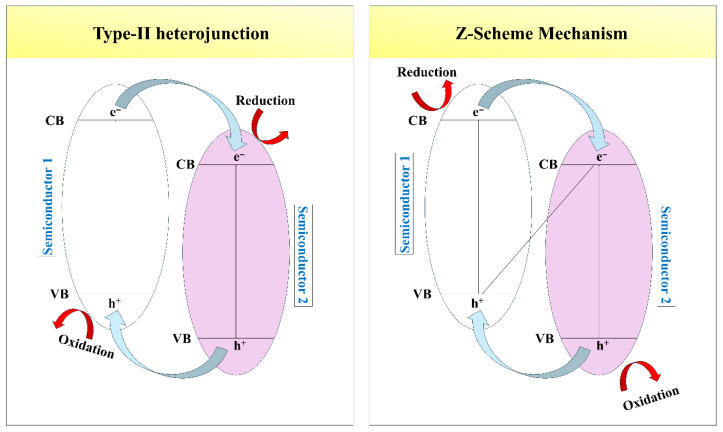
Schematic illustration of charge transfer pathways in type II heterojunction alignment and Z-Scheme photocatalytic systems.

Charge transport efficiencies and band engineering are key factors in determining the photocatalytic effectiveness of hybrid biopolymer–metal oxide systems, largely because they affect the separation and transfer of photogenerated electrons and holes. In hybrids, the right matching of energy levels between the biopolymer and metal oxide enables the charge carriers to be directed in a certain way, and thus the recombination of charges is minimized. Usually, when exposed to light, electrons gain energy and jump from the valence band to the conduction band of the semiconductor. Then, they travel through the interface to a conduction band or conductive path with a lower energy level, whereas holes move the other way, leading to spatial separation of charges [94]. If the energy levels of the biopolymer match those of the metal oxide, electrons can cross the interface more readily, and thus their lifetime and availability for photocatalytic reactions are increased [15]. One very effective technique to enhance the separation of charges in hybrid systems is type II band alignment, which creates a staggered band structure between the conduction and valence band edges of both components. This type of alignment drives electrons and holes to move towards different phases, which separates them effectively and suppresses recombination. This suppression gets even stronger due to internal electric fields that develop at the interface and produce directional charge migration, thereby lowering the probability of electron–hole recombination [95]. The Z-scheme charge transfer system is another type of mechanism that efficiently maintains strong redox ability and enables charge separation. This mechanism has been widely reported to enhance the photocatalytic activity of hybrid systems [96]. Type II heterojunctions cause photogenerated electrons and holes to be transported to distinct semiconductors with lower conduction bands and higher valence band energy levels, respectively, that enhance charge separation. However, this comes at the expense of overall redox potential [94]. Alternatively, the Z-scheme mechanism allows the recombination of electrons and holes with low energy levels while keeping the electrons and holes with high energy levels in their separate bands. Hence, strong reduction and oxidation powers are maintained [95]. This difference makes Z-scheme systems capable of achieving highly efficient reactions that require large redox potentials, even though both mechanisms lead to the enhancement of charge separation [94]. Band engineering strategies like defect creation, heteroatom doping, and surface functionalization are often applied to further increase charge transport. These methods introduce intermediate energy states or internal electric fields, promoting electron mobility and delaying charge recombination. These strategies not only improve charge carrier mobility but also enhance the lifetime of photogenerated electrons and holes, which is important for efficient surface redox reactions [97]. Furthermore, the presence of biopolymers provides continuous ionic or hydrogen-bonded pathways that support charge transfer at the interface [98]. In a review by Low et al. [61], properly engineered heterojunction photocatalysts are outlined to enhance the spatial separation of photogenerated charges and increase interfacial electron transfer through careful control of band alignment and interface structure, resulting in improved photocatalytic performance. As illustrated in Figure 6, in a type II heterojunction system, photogenerated electrons migrate from the conduction band of one semiconductor to that of another with lower energy, while holes move in the opposite direction, resulting in effective spatial charge separation. In contrast, the Z-scheme mechanism involves the recombination of low-energy electrons and holes at the interface while retaining high-energy charge carriers in their respective bands, thereby preserving strong redox potential for photocatalytic reactions [94]. The schematic diagram of the type II heterojunction and Z-scheme mechanisms in Figure 6 further explains the band alignment and charge transfer routes in these hybrid systems. Overall, rational band engineering and improved charge transport at the biopolymer–metal oxide interface are crucial for maximizing photocatalytic efficiency and minimizing recombination losses [93]. Therefore, the photocatalytic efficiency of these hybrid systems largely depends on the interplay between the composition of the materials, their band structure alignment, and the behavior of charge carrier transport.

## 7. Photocatalytic Applications

Semiconducting metal oxide and biopolymer–metal oxide hybrid systems possess practical photocatalytic performance toward the degradation of environmentally toxic pollutants. Synthetic dyes and pharmaceutical residues are two major categories among various contaminants due to their chemical stability, toxicity, and resistance to traditional treatment processes [19]. In addition to organic pollutants, the detoxification of heavy metals and antimicrobial applications of photocatalytic systems have also been explored [99]. Several parameters, like bandgap energy, light absorption capacity, charge carrier mobility, surface area, adsorption affinity, etc., govern the efficiency of photocatalytic degradation. Catalytic performance mainly depends on these structural properties of the photocatalyst. Structural characteristics of materials, such as porosity, surface groups, and heterojunction design, directly affect electronic properties such as band structure and charge carrier dynamics and naturally affect photocatalytic degradation efficiency [56]. Metal oxide semiconductors such as TiO_2_, ZnO, and doped oxide systems have demonstrated better degradation capability under UC and visible irradiation. However, modifications such as metal doping, rare-earth incorporation, and heterostructure formation show better performance because of better charge separation and band structure timing.

### 7.1. Degradation of Dye Pollutants

The application of synthetic dyes is an inherent part of various facets of life. These dyes or colored pollutions are highly complex, and to remove them is a big challenge for industries and researchers also. Despite the widespread benefits of the dyes, the presence of dye effluents in water is very harmful to life on Earth. Studies have found that the dye molecules like methylene blue (MB), malachite green (MG), Victoria blue R (VBR), methyl orange (MO) and direct red 5B (DR-5B) are usually used as typical model pollutants for photocatalytic activity determination [100]. In fact, metal oxides such as ZnO and TiO_2_ have the ability to produce a strong oxidation potential under UV light, which is helpful to degrade the dyed molecules efficiently through the generation of reactive oxygen species (ROS) such as hydroxyl radicals and superoxide anions. Reactive oxygen species are produced when photogenerated charge carriers engage with surface species; for example, conduction band electrons transform dissolved oxygen into superoxide radicals (•O_2_^−^). On the other hand, valence band holes can oxidize water or hydroxide ions to yield hydroxyl radicals (•OH). Both radicals are highly reactive in decomposing organic pollutants [101]. Linsebigler et al. [102] have given a detailed explanation of the mechanism of TiO_2_ heterogeneous photocatalysis. They showed that photogenerated charge carriers provide the driving force for redox reaction initiation, resulting in degradation of organic pollutants. As for ZnO, the work of Özgür et al. [55] analyzed how the intrinsic characteristics of the material contributed to its capability towards oxidative degradation of model dyes such as methylene blue. Regardless of their high activity under UV light, pristine oxides undergo rapid recombination of photogenerated electron–hole pairs. Diebold [103] highlighted surface defect states and charge trapping phenomena in TiO_2_, exhibiting the limited photocatalytic efficiency due to recombination at surface sites. This recombination loss leads to an effect in kinetics of dye degradation. To mitigate these limitations, noble-metal modification has been widely employed. Shuang et al. [104] demonstrated that Au–Pt co-decorated TiO_2_ nanopillar arrays led to the development of a synergistic electron-sink function property of Pt and localized surface plasmon resonance (LSPR) of Au, resulting in enhanced photocatalytic activity towards dye degradation. This synergism resulted in improved charge separation and increased visible-light absorption compared to bare TiO_2_. Correspondingly, Zheng et al. [105] prepared Au-decorated carbon-modified TiO_2_ nanocomposites that exhibited greatly enhanced visible light-driven photocatalytic activity for organic pollutant degradation, contributing to the improved light harvesting and charge separation facilitated by the plasmonic Au nanoparticles. Overall, these studies confirm the efficiency of metal loading in improving photocatalytic activity by extending light absorption into the visible range, promoting interfacial charge transfer and reducing recombination losses. As summarized in Table 2, various semiconductor-based photocatalysts demonstrate varying efficiencies for dye degradation on the basis of catalyst composition, reaction conditions, and irradiation time. The comparison indicates that catalyst modification and material selection play an important role in enhancing photocatalytic degradation efficiency and reducing reaction time for dye removal.

### 7.2. Degradation of Pharmaceutical Pollutants

**Figure 7:**
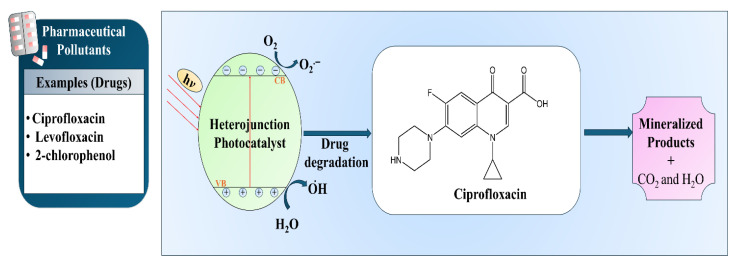
Schematic representation of visible light-driven photocatalytic degradation of pharmaceutical pollutant.

Due to the extensive use of pharmaceuticals, such as antibiotics, analgesics, and anti-inflammatory drugs, only a small portion of these drugs is removed during treatment in conventional wastewater treatment plants. Thus, pharmaceutical contaminants continue to be found in surface water and wastewater systems [108]. Ciprofloxacin, levofloxacin, and 2-chlorophenol are examples of such compounds that are both chemically stable and biologically persistent, which implies that they are potential sources of the development of antibiotic resistance and long-term ecological toxicity. These contaminants have complex aromatic structures that make them highly resistant to biodegradation, thus necessitating advanced oxidation processes like semiconductor photocatalysis [103,104]. Metal oxide semiconductors, especially TiO_2_ and ZnO, have demonstrated great potential in eliminating pharmaceutical pollutants through reactive oxygen species generation under light irradiation, as illustrated in Figure 7. Pelaez et al. [109] emphasized in their review that visible light-active TiO_2_ systems can degrade pharmaceuticals as well as other contaminants via radical-mediated oxidation pathways. Research shows that the degradation of pharmaceutical contaminants can be significantly enhanced by engineered heterostructured photocatalysts through increased visible-light absorption and the improvement of charge separation. In the work of Gan et al. [110], dual defect-modified 2D–2D (TiO_2_)g-C_3_N_4_ was identified as the photocatalyst leading to roughly 94.5% degradation of levofloxacin in 15 min under visible light due to better charge carrier separation and extended light response. Harikaran et al. [111] demonstrated ciprofloxacin degradation of around 99.8% in 30 min using a ZnO_2_–2D g-C_3_N_4_ heterojunction, and thus their work confirms the rationale behind the efficient interfacial charge transfer and reduced recombination of tailored semiconductors that in turn lead to rapid antibiotic removal. The above studies provide an insight: the rational synthesis of heterojunction and defect-rich semiconductor photocatalysts accelerates the degradation of pharmaceutical pollutants under visible light, which might be the key to the future of sustainable water remediation techniques. Even though these results emphasize the high removal percentages of the pollutants, they do not address whether the catalyst can be reused and how stable it is during the different cycles [6,112]. These two factors play the key role in determining whether the respective photocatalytic reactions are suitable for applications on a large scale outside the laboratory [112]. Specifically, biobased hybrid photocatalysts may undergo structural decomposition through repeated uses, including leaching of active components, disappearance of surface functional groups, or instability of the biopolymer matrix under prolonged irradiation, all of which may negatively impact the long-term performance of these materials [113].

### 7.3. Photocatalytic Reduction of Heavy Metal Ions

**Figure 8:**
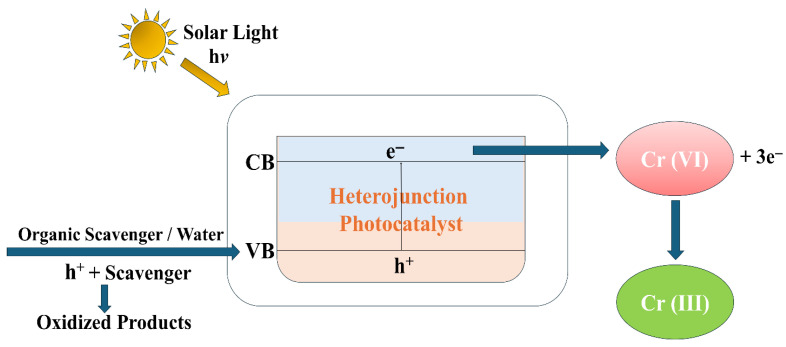
Schematic illustration of photocatalytic reduction of Cr (VI) over a heterojunction photocatalyst.

Photocatalytic heavy metal ion reduction has attracted growing attention as a green process for detoxifying metal species through their transformation to less harmful elements. Out of various heavy metals, Cr (VI) has been extensively researched because of its extreme toxicity and abundance in industrial wastewaters. Ghafoor et al. [114] achieved ~90% photocatalytic degradation of Cr (VI) using Ag_2_S-sensitized TiO_2_ nanofibers at acidic pH under solar irradiation. Similarly, Zahid et al. [115] used Ag–TiO_2_ photocatalysts to reduce Cr (VI) to Cr (III) under solar light. The photocatalytic reduction performance was strongly influenced by surface morphology and Ag loading, showing that noble-metal integration can enhance redox activity through plasmonic and electron-sink effects. Photocatalytic reduction mechanisms typically involve photogenerated electrons in the conduction band reducing metal ions, while the corresponding holes are consumed by an organic scavenger or agent, as shown in Figure 8. Ashar et al. [116] synthesized ZnO–CuO nanocomposite systems for the solar-driven reduction of Cr (VI) to the less toxic Cr (III). In this research, 97% of Cr (VI) was removed from wastewater in acidic media, highlighting the practical efficiency of these nanocomposites. In a study by Vavilapalli et al. [117], MX-ene–Ti_3_C_2_-based heterostructured composites were utilized for the efficient reduction of Cr (VI) by enhancing the generation of photocarriers and their separation. These studies have illustrated that band engineering, heterojunction formation, and surface modification are leading strategies in promoting the photocatalytic reduction of heavy metals, facilitating both environmental remediation and the potential recovery of valuable metals.

### 7.4. Antibacterial and Antimicrobial Photocatalytic Applications

Photocatalytic systems also exhibit antibacterial and antimicrobial properties, primarily driven by the generation of reactive oxygen species (ROS) under light irradiation, which can damage the cellular components of microbes. In multifunctional nanocomposites like Ag_2_S–TiO_2_, photocatalysts also demonstrate strong bactericidal effects along with Cr (VI) reduction, involving the complete inactivation of *E. coli* and high inactivation of *S. aureus* after 1 h under stimulated solar light, due to the synergistic effects of ROS and metal ion release [107]. In several studies, composite systems were engineered specifically to increase antimicrobial performance. Zyoud et al. [118] synthesized Fe-doped ZnO sub-microparticles via a laser-assisted method, exhibiting superior photocatalytic degradation of pharmaceutical contaminants and showing high antibacterial efficiency against *E. coli*, *S. aureus*, *K. pneumoniae*, and *C. albicans*. This highlighted that transition metal doping can narrow the bandgap and increase ROS production for biological inactivation. One study by Verma et al. [119] involving TiO_2_–ZnO–graphene oxide nanocomposites also highlighted the potential of hybrid systems that combine a semiconductor photocatalyst with a conductive carbon-based material leading to enhanced photocatalytic and antimicrobial activities against Gram-positive and Gram-negative bacteria. ROS production, including hydroxyl radicals and superoxide anions that stay at the photocatalyst surfaces under light irradiation, are capable of damaging bacterial membranes, proteins, and DNA, thus resulting in the loss of the membrane and cell death. This lowers the chance of resistance development when compared to the use of standard antibiotics [120].

### 7.5. Comparative Analysis of Biobased Hybrid and Conventional Photocatalysts

Most traditional inorganic semiconductor photocatalysts like TiO_2_ and ZnO are well known to have very high photocatalytic efficiency, especially when exposed to UV light [15]. However, their real-world use gets hindered to a great extent by factors like the quick recombination of charges, being incapable of absorbing visible light, and higher production costs, especially when modified with techniques like doping with noble metals [45]. On the other hand, biobased hybrid photocatalytic systems, due to the benefits of synergistic interaction between semiconductors and biopolymers, show better charge separation efficiency, stronger adsorption capacity, greater visible-light activity, and cost-effective accessibility, since biopolymers are typically abundant, renewable, and friendly to the environment [121]. Based on sustainability, economical production, and the feature of being compatible with the environment, biobased hybrid materials can even out their drawbacks in stability over a long period by offering real and practical benefits; thus, they can be regarded as viable substitutes for traditional inorganic photocatalysts when it comes to large-scale applications [66,114]. Conventional photocatalysts generally have high activity, while biohybrid systems have been shown to have higher pollutant adsorption capacity, enhanced utilization of visible light, and superior interfacial charge transfer. However, their long-term stability, reproducibility and scalability issues need to be properly tackled before their application at a larger scale [97]. Strategies like surface modification, cross-linking of biopolymers, and design of stable heterojunction architectures may be used to improve the longevity and efficient recovery of the catalysts for multiple cycles, thus helping to resolve the mentioned problems [120]. In this context, the integration of advanced design approaches, including hierarchical porous architectures and multifunctional hybrid nanocomposites, may significantly improve the performance, longevity, and ability to scale biobased photocatalytic systems.

## 8. Challenges and Future Prospects

Even though the biobased hybrid photocatalytic systems demonstrate substantial progress, they show several limitations, leading to challenges in transitioning these materials from the laboratory to real-world technologies. One of the major limitations is the stability of the materials. Many advanced photocatalysts, including those combining biopolymers with metal oxides, can undergo photocorrosion, structural degradation, or decomposition under prolonged light exposure or in harsh environmental conditions. These factors reduce the long-term effectiveness of these materials and raise concerns about secondary pollution like metal contamination during reuse cycles [120]. Reusability is another critical factor affecting the applicability of photocatalysts. Usually, in practical scenarios, these materials are expected to be efficient over a number of cycles; however, many photocatalysts tend to show a reduction in their activities gradually, due to fouling of the surface, loss of active sites, or changes in the structure when used repeatedly. Difficulties in separating and recovering photocatalysts from treated systems frequently prevent their reuse, and the overall efficiency of the process gets lowered. This leads to an increase in the operational cost and environmental risk. This stresses the need for creating photocatalysts that have better reusability [122]. Additionally, reproducibility across batches for synthesis remains limited, particularly for green and biologically derived materials, where natural feedstock variation leads to inconsistent properties and performance. Also, many laboratory studies report high photocatalytic efficiency under ideal conditions, but real wastewater systems are mixed with contaminants and have fluctuating pH and high turbidity. These factors reduce the activity, underscoring the need for proper evaluation of hybrid materials in complex systems [123]. Besides these factors, real wastewater is a very complex mixture of various types of organic and inorganic pollutants, competing ions, and suspended solids that can greatly affect photocatalytic reactions, not only by blocking the opening for the active part but also by making it difficult for light to reach [46,62]. One major issue that prevents wider use of photocatalysis is catalyst recovery, especially for nanoparticle-based schemes, where separation from treated water is difficult and may result in secondary contamination [108]. Also, long-term use without maintenance is a critical issue, as it can cause damage to component structure, leaching of active parts, and a decrease in efficiency. All these problems clearly indicate the need for a new photocatalyst system that is not only highly efficient and recoverable but also stable even under severe environmental conditions [81]. Another significant barrier includes scalability and environmental sustainability. Scaling up synthesis methods from laboratory synthesis to industrial production often indicates issues, like fabrication routes, high costs, and challenges in controlling morphology and consistency over large batches [60]. Several scalable emerging fabrication techniques such as continuous flow synthesis, large-scale sol–gel processing, hydrothermal synthesis in industrial reactors, and advanced material structuring approaches are currently under exploration for the manufacturing of biobased hybrid photocatalysts [49,65]. These techniques provide better control over reaction conditions, better reproducibility, and higher potential for large-scale production, thus facilitating the shift from lab research to industrial use [124]. For green and bioinspired photocatalysts, the variation in natural precursors, potential supply limitations of biomass sources, and lack of large-scale protocols complicate the commercial production of these materials. Comprehensive long-term environmental impact studies and biodegradation analysis of hybrid photocatalysts are still limited, which limits the confirmation of the applicability of these sustainable approaches compared to conventional pollutants and treatment technologies [125]. For future advancements, emerging trends such as bioinspired materials, green fabrication techniques, etc., present exciting opportunities to overcome existing limitations. For instance, bioinspired synthesis routes can reduce dependence on toxic chemicals while promoting structural stability and accelerate discovery and performance of these materials by optimizing compositions and predicting long-term behavior across different environmental conditions [126]. Future work should also aim at upgrading the structural stability and recyclability of biobased photocatalysts by implementing measures like surface modifications, cross-linking of biopolymer matrices, and creating stable heterojunction systems to guarantee their stability over long periods.

### Emerging Research Directions

The area of biobased hybrid semiconductor photocatalysts is rapidly evolving, with multiple research directions gaining significant attention. One major shift has been the development of solar-powered photocatalytic systems capable of efficiently using visible light, which improves energy efficiency and leads to sustainability [127,128]. Besides that, eco-friendly and scalable green synthesis methods, such as bioassisted and low-energy fabrication processes, are being investigated for environmentally friendly and large-scale production [129]. The adoption of bioderived semiconductor materials from renewable resources is another route attracting attention because of their low price, availability, and tunable properties [130]. Furthermore, the design of multifunctional hybrid materials with additional properties such as antimicrobial, adsorption, sensing functions, etc., is becoming a trend and providing combined environmental applications. These improvements highlight the potential for developing efficient, sustainable, and application-oriented photocatalytic systems in the future [93,127].

## 9. Conclusions

Recent advances in biobased hybrid semiconducting photocatalysts have demonstrated considerable improvements in environmental remediation, multifunctionality, and sustainability. The integration of renewable biopolymers with metal oxide semiconductors at the molecular level has enhanced charge separation efficiency, modulated electron–hole dynamics, and broadened light absorption, effectively addressing the limitations of traditional inorganic catalysts. These molecularly engineered hybrids facilitate the efficient degradation of organic pollutants, reduction of heavy metals, and antimicrobial activity.

Innovative design strategies, including in situ synthesis, heterojunction engineering, and surface functionalization, allow precise molecular control over the interface between polymeric and inorganic phases, directly improving photocatalytic performance and versatility across diverse media.

For future directions, integrating sustainable, high-performance hybrid materials into real-world applications will require overcoming limitations such as long-term stability, scalable synthesis, and environmental compatibility. Continued research should emphasize eco-friendly synthesis routes, reproducible material fabrication, and mechanistic understanding of molecular-level charge transfer and interfacial interactions.

Additionally, future investigations might benefit from interdisciplinary approaches, for example, combining biobased hybrid photocatalysts with membrane filtration systems, adsorption technologies, or advanced reactor designs for wastewater treatment. These cross-field strategies could not only lead to higher removal efficiency of pollutants but also allow for continuous operation and make the materials more practically applicable in real environmental systems. Overall, molecular-level engineering of hybrid semiconductors will be key to bridging the gap between laboratory research and practical implementation, enabling environmentally compatible, highly efficient photocatalytic systems.

## Figures and Tables

**Table 1:** Comparative properties, advantages and limitations of commonly used metal oxide semiconductors.

Semiconductor	Type	Bandgap (eV)	Light Response	Key Advantages	Major Limitations	Reference
ZnO	n-type	~3.3	UV-responsive	High exciton binding energy; strong oxidative capability; good intrinsic optical and electronic properties	Rapid charge recombination	[55]
TiO_2_	n-type	~3.0–3.2	UV-responsive	High structural stability; non-toxic; widely studied crystalline forms	Limited visible-light absorption; charge recombination and adsorption issues	[56]
SnO_2_	n-type	~3.6	UV-responsive	Excellent chemical stability; high electron mobility	Wide bandgap limits visible-light absorption	[57]
α-Fe_2_O_3_ (hematite)	n-type	~2.1	Visible-light active	Suitable bandgap; promising for pollutant degradation and solar water oxidation	Short hole diffusion length; rapid charge recombination	[58]
CuO	p-type	~1.2–1.7	Visible-light active	Narrow bandgap; better visible-light absorption	Charge recombination reduces photocatalytic efficiency	[60]

**Table 2:** Photocatalytic degradation of some commonly used dyes under different catalysts, light sources, and modification approaches.

Dye Pollutant	Catalyst Used	Synthesis/Modification	Light Source	Degradation Time (Minutes)	% Degradation	Reference
Methylene blue	ZnO	Pristine semiconductor	UV light	150	94	[106]
Malachite green	AuNPs	Plasmonic metal modification	Visible light	100	81	[43]
Victoria blue R	TiO_2_	Pristine semiconductor	UV light	720	50	[100]
Methyl orange	Pt-doped TiO_2_	Metal doping	UV-visible	90	98	[107]
Direct red 5B	Ag-doped TiO_2_	Metal doping	UV-visible	90	90	[82]

## Data Availability

No new data were created or analyzed in this study. Data sharing is not applicable to this article.

## Abbreviations

Ag	silver
Ag_2_S	silver sulfide
Au	gold
CVD	chemical vapor deposition
CuO	copper (II) oxide
Cr (VI)	hexavalent chromium
Cr (III)	trivalent chromium
Fe_2_O_3_	ferric oxide
g-C_3_N_4_	graphitic carbon nitride
GO	graphene oxide
H_2_O_2_	hydrogen peroxide
LbL	layer by layer
LSPR	localized surface plasmon resonance
MXene	two-dimensional transition metal carbides/nitrides
NCC	nanocrystalline cellulose
Pt	platinum
ROS	reactive oxygen species
SnO_2_	tin (IV) oxide
TiO_2_	titanium oxide
UC	ultraviolet C
UV	ultraviolet
ZnO	zinc oxide
